# Investigating how COVID‐19 has challenged the Eurocentric concept of ‘development’: a case for sustainable food systems in the UK

**DOI:** 10.1002/fes3.416

**Published:** 2022-08-23

**Authors:** Eniola Shittu, Komali Kantamaneni, Luiza C. Campos

**Affiliations:** ^1^ University College London London UK; ^2^ Faculty of Science and Technology University of Central Lancashire Preston UK

**Keywords:** food supply chains, food system, pandemic, development, sustainability, UK COVID‐19

## Abstract

The COVID‐19 health crisis has imposed extensive shocks to many global systems, particularly the UK food production chains, further challenging Eurocentric development discourses and stereotypes. Thus, this paper investigates how the pandemic has challenged the UK's development status by analysing how the pandemic has impacted the country's food industry. A literature review was conducted and used to identify, select and critically appraise publications between 2000 and 2021 discussing the challenges in the UK food system. The findings reveal that the UK's food industry is unsustainable as there are significant flaws in the system, that is food insecurity and food waste that go unaddressed. The impact of the pandemic has exacerbated the social and economic impacts of operating with such a system. Compounded with the geopolitical adjustments caused by Brexit, the UK is faced with the challenge of restructuring and developing new frameworks such as policies, regulations, schemes and partnerships to support the food industry's sustainability. Lastly, the findings reinforce that ‘developed’ and ‘developing’ nations encounter similar food challenges, which manifest differently in various landscapes and contexts. Therefore, the world (not just the UK) needs to shift away from Eurocentrism, moving towards a universal but equally personalised development outlook. This review provides an outline of the major problem areas in the UK food system and presents potential solutions aimed at helping guide the government's decision‐making process.

## Graphical abstract

The COVID‐19 pandemic reinforced the reality that the UK's Food system is unsustainable The findings provide a unique insight on the UK food system from a Global development lens. Bridges the gap between pre‐pandemic literature and emerging post‐pandemic research. The research encourages new innovative ways to conceptualise and measure Development.

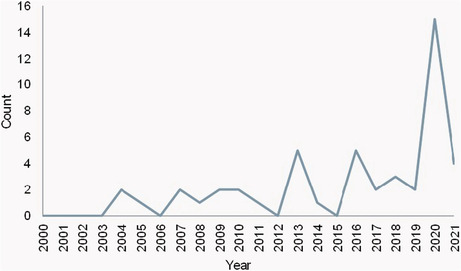

## 1 INTRODUCTION

Development studies is a multidisciplinary subject, which focuses on the advancement of nations from a political, cultural, geographical and socio‐economic lens. To ask, ‘What is development studies?’ begs the question ‘What is development?’ (Leach et al., 2021). Although there are multiple interpretations and comprehensions of the term, it is typically perceived to be intimately intertwined with Eurocentrism. Leach et al. (2021) describes development as the pursuit of modernisation through ‘the transfer of knowledge and resources from “developed” to developing nations’. Researchers like Raworth (2018) have questioned the relevance of development signifiers in contemporary studies, highlighting that the increasing poverty and inequality amongst other socio‐economic and environmental problems are becoming more visible in the Global North; thus, he argues that development applies to everyone everywhere. The pandemic has raised many questions regarding conventional development discourse, practices and principles, thus requiring us to reframe our comprehension and approach to development.

The sustainable development goals (SDGs) reflect a collaborative effort to tackle core challenges threatening human well‐being, preventing economic prosperity and causing environmental degradation (Pradhan et al., 2017). Moreover, SDGs represent a universal acknowledgement that all countries require development to varying extents (Osborn et al., 2015). However, Vries (2020) discusses the impact of culture on the attainment of the SDGs, explaining that culture should be regarded as one of the pillars of development as it plays a significant role in the approach taken to attain development. Western Europe and the United States are used as blueprints for social, economic and political development (referred to as Eurocentrism). Consequently, the romanticisation of Eurocentrism resulted in the disruption of the natural trajectory of development of countries in the Orient (Segage, 2018).

In addition, Brohman (1995) explains that Eurocentrism has permeated modern‐day frameworks in development studies. Consequently, the cultural superiority of the Western world still lines the foundation of contemporary social and economic structures, and its influences continue to encroach on Eastern progression. Remnants of Eurocentrism are still seen in the way intergovernmental organisations categorise countries. For example, developed countries refer to a sovereign state with a highly prosperous economy and considerable technological infrastructure, while developing nations have low industrialisation and human development index (Surbhi, 2020). The pandemic has caused a shift in the development paradigm. Casti (2012) believed that complex systems in society are vulnerable to X‐events (unexpected events) as they cause abrupt changes in the system. The occurrence of the pandemic has raised a critical eye to conventional social and economic systems as the pandemic exposed the fragility within these structures. There has been a surge of pandemic/postpandemic literature, which focuses on the impact of COVID‐19 in various dimensions and facets of society including global health and the economy. However, there is limited research, which investigates the impact of the pandemic on food systems.

The UK is perceived as one of the world's most developed countries. However, reports reveal that the country's food system has grappled with the level of disruption resulting from the pandemic; the Guardian describes the severity of this problem as a 'food crisis' (Laville, 2020). The repercussion of the virus has been far‐reaching on multiple levels in the food industry (Laville, 2020) and is a concern amongst the public and experts. Therefore, the aim of this work was to investigate how the occurrence of the COVID‐19 pandemic has challenged the UK's development status and impacted the country's food supply chain. This aim was achieved by three specific objectives: (i) identify the challenges existing in the UK food system between 2000 and 2021; (ii) discuss how the pandemic has exacerbated pre‐existing or created new obstacles in the food system and lastly (iii) provide solutions to help improve the UK agri‐food sector, particularly how to adjust policies and practices to better align with the standards set by the SDGs.

## 2 METHOD

A comprehensive literature review was conducted on the challenges (i.e. relating to food supply, distribution and production) in the UK food system.

### 2.1 Eligibility criteria

The criteria for inclusion were studies that have been published between 2000 and 2021 and must be focussed on the UK. Documents had to be published in English or have an English‐translated version available and accessible. There were no restrictions on the type of publications, that is thesis, reports, journal articles or grey literature eligible for selection. All literature included were published and accessible, either through online sources or through physical forms. Literature that do not comply with these criteria (see Table 1) were excluded during the screening process.

**TABLE 1:** Eligibility criteria for included documents according to the population‐ exposure‐ outcome‐ study (PEOS) design framework

Inclusion	Exclusion
UK focus	Not focussed enough on the UK
Published between the years 2000 and 2021	Literature not published in English
Any type of literature, that is blogs, journal articles and reports	Not accessible physically/online
Relevant to the scope of the research	Literature found from social‐networking platforms and site forums
Literature relevant to the UK food system	–

### 2.2 Sourcing, searching and selection

An extensive literature search was performed using Google Scholar, Scopus and Web of Science. These databases were chosen as they are classic and well‐known academic search engines; hence, these platforms are recognised for providing credible sources. The search terms used in the electronic databases were ‘UK food supply’, ‘UK food distribution’, ‘food production’ and ‘UK food system’. These keywords are relevant to the topic of the review and represent the type of literature aimed to be collected in the search. The phrases were combined using the Boolean operator, which searched the phrases in the titles, abstract and keyword of every published document in three databases.

Before embarking on the screening process, duplicates were removed from the total records obtained. During the screening process, the quality, relevance and full‐text accessibility of the literature were checked using the preferred reporting items for systematic reviews and meta‐analyses (PRISMA) checklist and inclusion criteria. Literature that was irrelevant, inaccessible, low quality, where the title was unclear, did not represent the scope of the research topic, or had an abstract that did not clearly describe the aim, objectives, findings and conclusions, were excluded. Not every literature screened had an abstract; in those cases, the introduction and conclusion were assessed instead. A full‐text assessment was conducted during the eligibility stage to ensure the paper corroborates with the theme and context of the review.

The PRISMA checklist was used to check the quality of the analysis of each document. It is acknowledged that the turbulent social conditions caused by the COVID‐19 pandemic may have impaired the quality of the literature written in the past 2 years. Therefore, these literatures may not reflect the publishing standards prior to the pandemic. Despite this, the content was still deemed relevant and necessary to include and discuss in the review. There were no restrictions to the types of literature included, as some grey literature has been included despite PRISMA categorising them as a less critical and less quality than traditional academic literature. It was crucial to include grey literature as the content discussed is relevant and may support academic debates relating to the dominant topic of this paper.

### 2.3 Data extraction and synthesis of findings

Data were extracted from each included literature and entered into a preformatted spreadsheet. The following information was extracted from each document: document type, authors, year of publication, title, journal, language, aim and objectives, and study design. Furthermore, the themes and the key issues raised in each literature were summarised and categorised.

## 3 RESULTS

**FIGURE 1:**
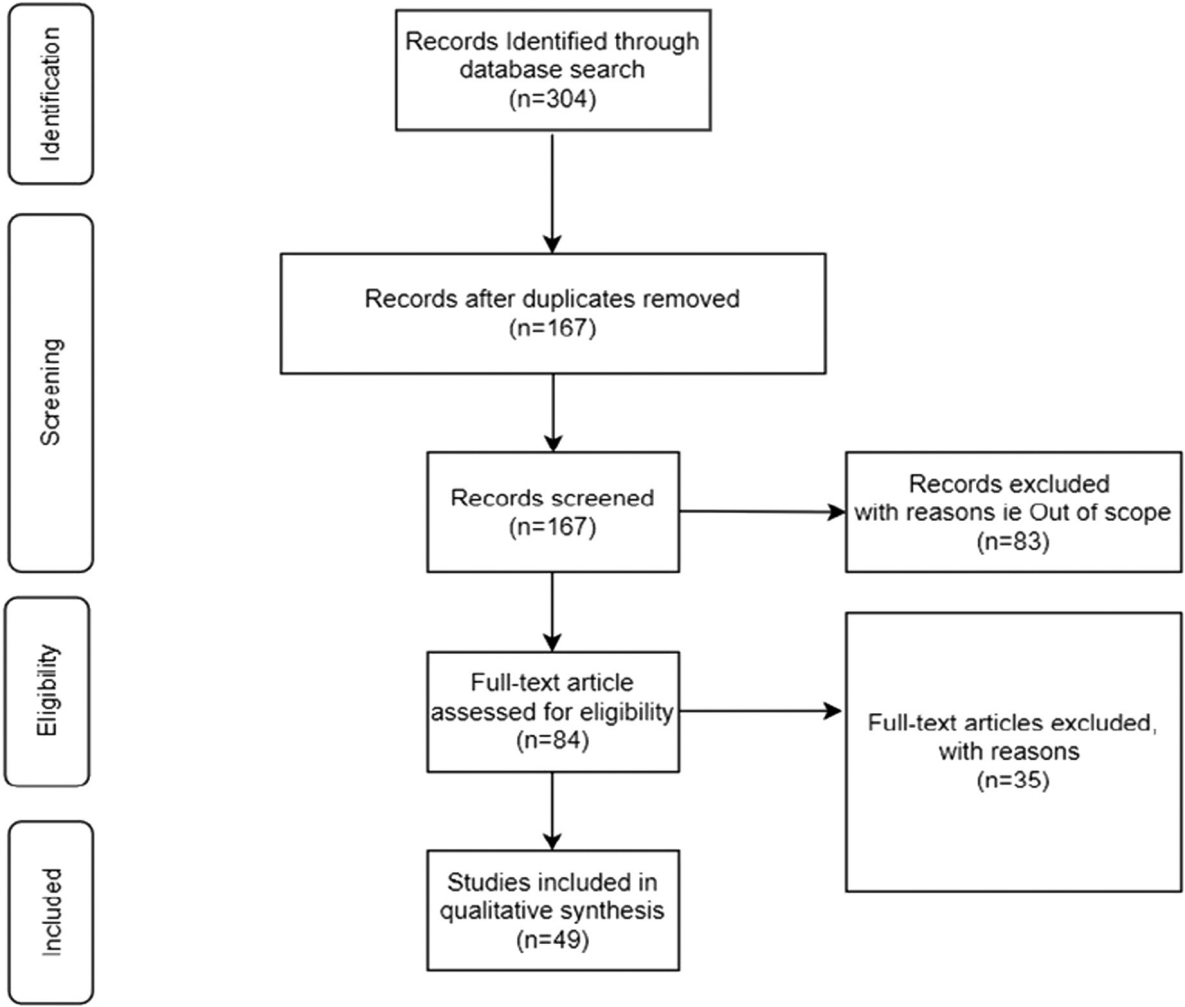
PRISMA flow chart visualising process (modified from Moher et al. (2009))

**FIGURE 2:**
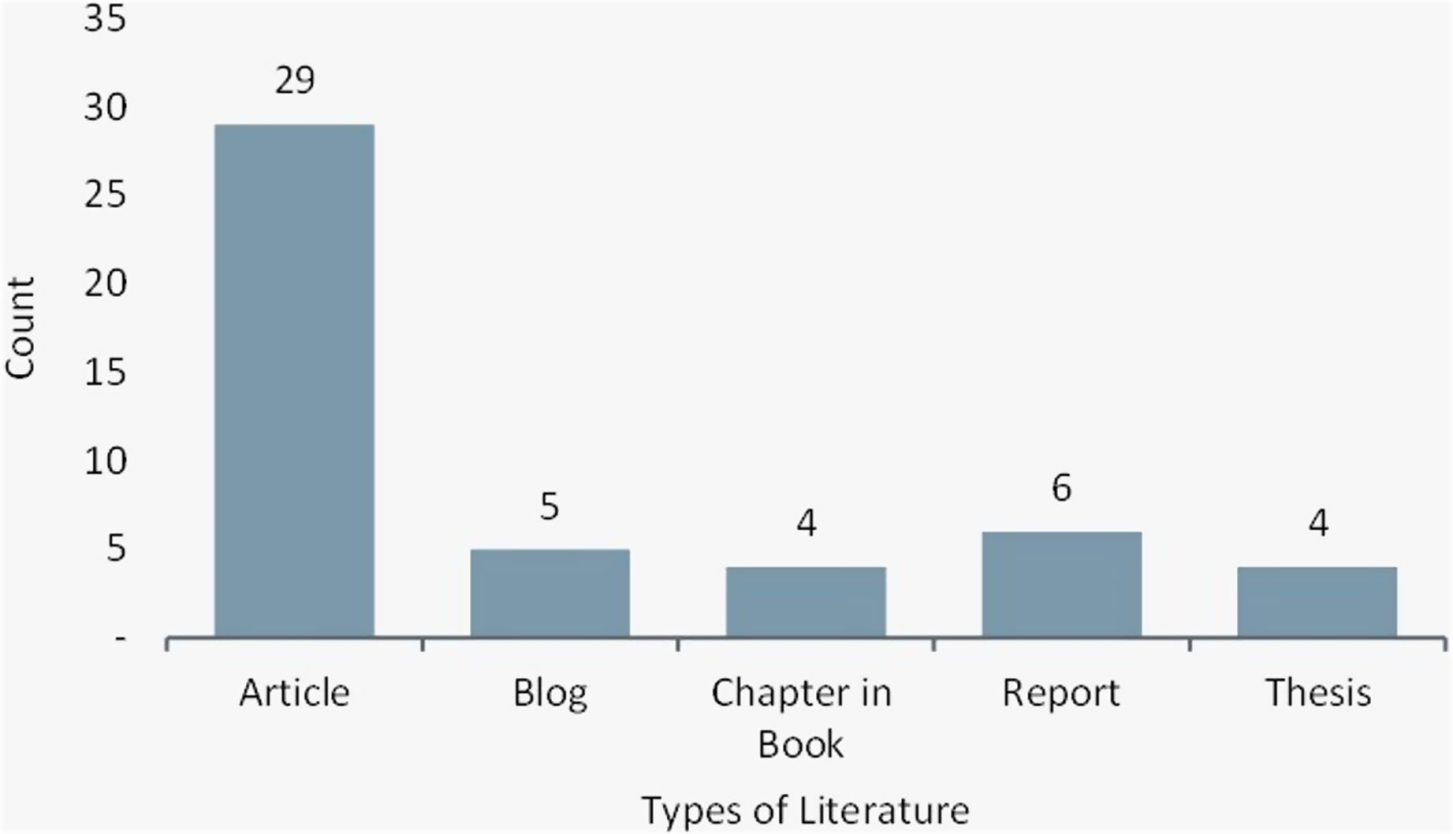
Types of publications

**FIGURE 3:**
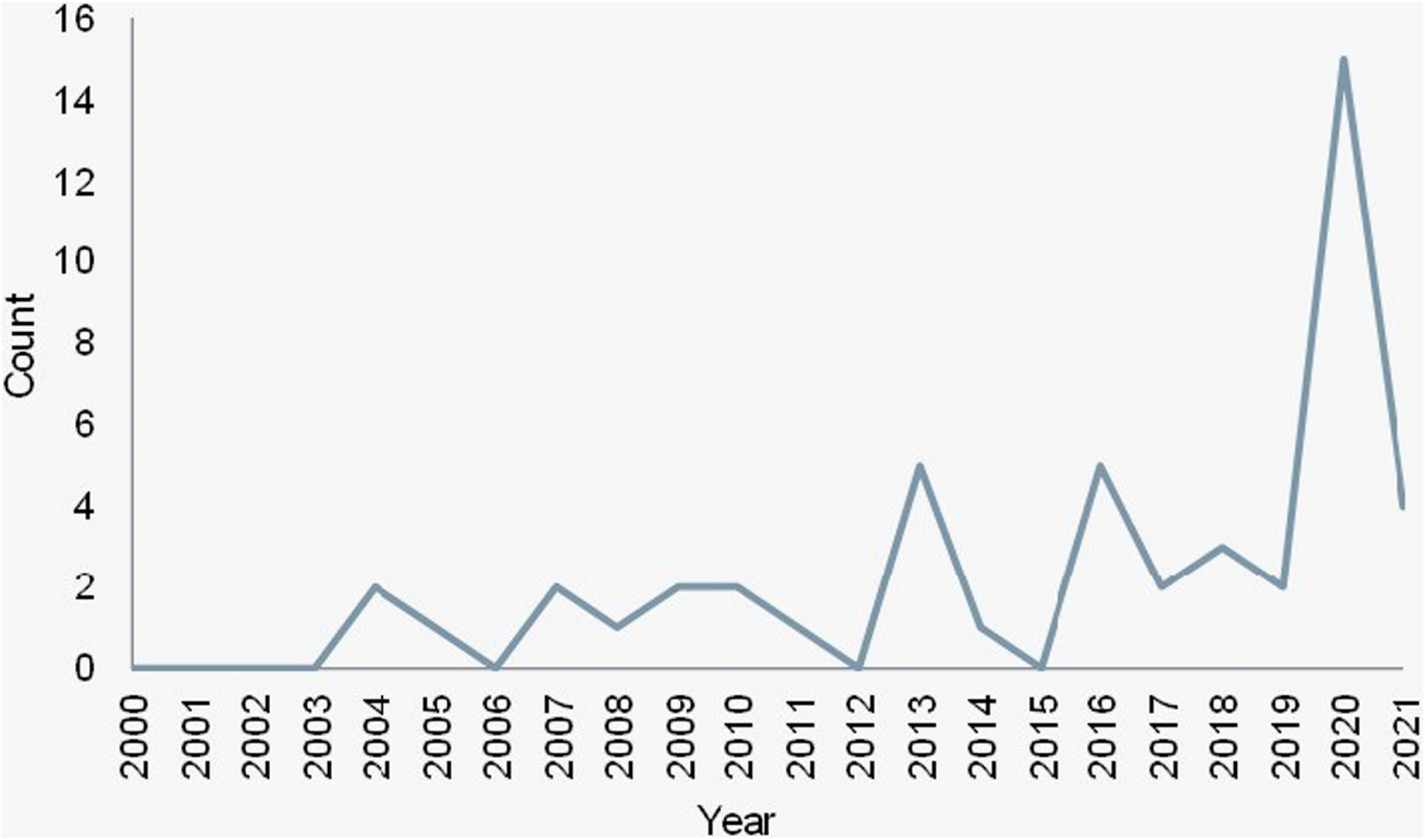
Year of publication (2000–2021)

During the identification stage of the process, 304 pieces of literature were identified from the different databases. After removing duplicate publications, there were 167 pieces of literature left to screen. 83 publications were excluded as they were beyond the scope of this comprehensive review. The full‐text screening was conducted, and 49 papers were eligible for inclusion in the review, and 35 were excluded for accessibility reasons (Figure 1). The comprehensive review included 49 different pieces of literature; 59% are journal articles (see Figure 2). This outcome suggests that discussions on the challenges existing in the UK food system are primarily discussed in academia. Also, the publications spanned the years 2000–2021, with the most significant number of literature being published in 2020; this may be due to the impact of the pandemic on the country's food supply chain (Figure 3). Observations show that less literature were published about food system challenges in the early 21st century (see Table 2), with slight variations caused by changes in social, economic and political conditions.

**TABLE 2:** Challenges in the UK food supply chain

Focus	References	Key issues raised
1. Food insecurity	Barling et al. (2010), Bhunnoo and Poppy (2020), Karki, Bennett and Mishra (2021), Lee and Marsden (2011), Power et al. (2020), Price (2020), Tomlinson (2013), Tsolakis and Srai (2017)	Governments and Defra's lack of prioritisation of food security. Poor access to nutritional food in the UK due to socio‐economic, cultural, and biological factors. The just‐in‐time operations in the food system cause challenges and concerns for food. COVID‐19 causing more barriers to accessing food for different groups of consumers.
2. Systematic failures (risk/resilience/uncertainty/vulnerability)	Bailey (2016), Elliott and Bhunnoo (2021), Ingram et al. (2020), Mitchell et al. (2020), Moran et al. (2020), Do et al. (2021)	National food and nutrition security are sensitive to volatility in the global food market. Cities' dependence on global resources has made them highly vulnerable to shocks that can disrupt the current supply systems. Localised supply chains although desirable for achieving sustainability, may not meet consumer preferences or reduce supply chain vulnerability.
3. Sustainability	Ghadge et al. (2020), Holmes (2018), Lee and Marsden (2011), Lee‐Woolf (2009), Yakovleva (2007)	The dairy industry faces particular challenges preventing the adaptation of sustainable practices in operations, with different size enterprises facing different barriers. The food supply chain in the UK's underperformance in terms of economic outputs: profitability and productivity is low.
4. Challenges in local and regional/urban and rural systems	Dubbeling et al. (2016), Maye and Kirwan (2013), Lee‐Woolf (2009), Oglethorpe and Heron (2013), Schmutz et al. (2017)	Lack of inclusivity in distribution networks is a leading constraint on local producers presence in the sector. Government strategies and approaches overlook the contributions and significance of local supply systems in attaining food security.
5. External factors	O'Carroll (2019), Ridler (2017), Tranter et al. (2007)	Concerns about the UK government's ability to establish effective food and safety regulations after Brexit. EU's lack of science‐based decision‐making regarding pesticide regulations.
6. Consumers	Armstrong and Reynolds (2020), Draper et al. (2019), O'Keefe et al. (2016)	Consumers' reliance on food safety labels in determining quality reinforces the importance of the UK establishing clear communications of post‐Brexit food regulations. Consumers are resistant to sudden changes to food policies, hence creating barriers to implementing new regulations and policies.
7. Food waste	Cao et al. (2020), Thapa Karki et al. (2021), Mena et al. (2014)	UK food manufacturing supply chain (FMSC) members lack knowledge on increasing and creating collaboration opportunities with other members to help reduce waste. Actors' failure to recognise the value of surplus food.
8. Social, economic and environmental impact	Aikins and Ramanathan (2020), Allinson (2004), Audsley et al. (2009), Dicks et al. (2013), Ruiter et al. (2016), Wilshaw (2018), Barling (2020), Harvey (2020), Henderson (2021), Shanks et al. (2020), Cummins et al. (2020)	The UK's dependence on international trade contributes to the increased emission of greenhouse gases. Britain's reliance on EU temporary labourers to provide a large labour force for the agricultural sector. Lack of prioritisation of public health in the proposed Agricultural Bill 2019–21.
9. Research and development	Ingram et al. (2013), Kirwan (2004), Leaver (2010), Li et al. (2020), Leat and Revoredo‐Giha (2008)	A leading concern for consumers, preventing their access to nutritious food is affordability. Lack of research and literature on the UK food sector. Lack of financial support from the government for research and development in the agricultural sector.
10. Systemic structure	Hingley (2005), Hingley et al. (2016)	Unequal power dynamics in the UK food system, that is the dominance of supermarkets in food supply chains. Specialist suppliers encounter economic barriers which prevents the development of long‐term relationships with buyers.

Abbreviation: Defra, Department of Environment, Food and Rural Affairs.

### 3.1 Characteristics of the documents included

According to Tansey and Worsley (2014), a food system is linked to three aspects of life: (i) biological processes which produce food and ecology within the biosphere, (ii) economic systems and political institutions and lastly, (iii) social and cultural aspects of life where food fosters social context and represent cultural traditions (Tansey & Worsley, 2014). Thus, the food system is essential to all facets of life as it helps these three areas interact and interlink with one another. However, recently, the sustainability of the country's food system has been questioned as the pandemic has increased the visibility of problems in the system by exacerbating existing issues and creating new ones. These challenges will be discussed in three parts: prepandemic, pandemic and re‐occurring challenges.

#### 3.1.1 Prepandemic

##### Systemic structure

In the early 21st century, the systemic structures impacted relationships between different stakeholders, especially in food procurement. Hingley (2005) discusses the dominance of supermarkets in the food supply chain in the UK. He recognised that power imbalances favour large‐scale retailers, whereas producers generally encounter higher risks, explaining that the power imbalance is inherent to supply chains. Hingley later collaborated with other researchers by interviewing intermediaries about the challenges of improving specialist supplier relationships with buyers (Hingley et al., 2016). They found that large retailers generally lack the economic motivation and flexibility to engage directly with small and niche suppliers (Hingley et al., 2016); therefore, small suppliers are left reliant on intermediary partners in helping to facilitate sales. The involvement of intermediary organisations in helping to bridge the gap in communication between suppliers and buyers is prominently observed in fresh food supply chains in the UK (Hingley et al., 2016).

Moreover, Hingley et al. (2016) discuss how some small and specialist suppliers have adapted to living with the power imbalance, that is forming alliances with mainstream suppliers. It is essential to consider that the responses given by the intermediaries are subjective; although their opinions may contain aspects of truth, they may also be biased. Also, it must be highlighted that intermediaries' suggestion for specialist suppliers to accept their submissive position in the supply chains can be perceived as an inherent response to protect their interests. In the same way, small suppliers rely on the services provided by the intermediaries, equally, intermediaries exist because of the imbalance; their role in the chain would be compromised without this issue. Furthermore, this suggestion reveals the oversimplified comprehension of the impact that imbalanced power dynamic in food supply chains (FSCs) can have on small suppliers, as Glavee‐Geo et al. (2021) research later reveals that power imbalance leads to the exploitation of the weaker party.

Although Hingley et al. (2016) work demonstrates how power dynamics can inhibit the development of relationships between specialist suppliers and large retailers, their findings do not provide a holistic insight into the obstacles preventing partnerships in the FSCs. Their research solely focuses on the perspectives of intermediaries; thus, it negates the perspectives of small suppliers and large retailers (Hingley et al., 2016); further research is necessary to gain the insight into retailers and small suppliers. Resolving such problem is vital as Jarzębowski et al. (2020) reinforces the importance of small suppliers in establishing shorter more sustainable FSCs.

##### Sustainability

Aside from the imbalanced systemic structure, the UK food system also struggles with a lack of sustainability; this concern has been widely acknowledged in academic and grey literature. Yakovleva's (2007) recognised that the UK's FSCs were unsustainable, particularly the chicken and potato supply chains, as both rely on imports at various stages in their chain. The agricultural industry in the UK suffers from severe price pressures as farmers compete with international prices of produce. Consequently, Yakovleva (2007) explains that low productivity, particularly in the British chicken industry, is not only unprofitable but also has long‐term detrimental environmental effects because of the reliance on imports. Yakovleva (2007) focuses on the chicken and potato supply chains; therefore, her findings may not reflect the entire industry. However, when the paper was published, potatoes and chicken were staples and represented two important groups of products in the UK: a popular protein and carbohydrate sources. It is likely that Yakovleva's (2007) findings reveal a lot about the state of the country's food system as fundamental issues existed in the two major supply chains in the country. Moreover, contemporary publications such as Holmes (2018) and Ghadge et al. (2020) support the earlier argument and findings of Yakovleva (2007) as they similarly recognise the unsustainability of the UK's beef, bean and dairy production line.

Lee‐Woolf (2009) explains that the UK's food system is not sustainable as it has had an adverse socio‐economic and environmental impact on a domestic and global scale. For example, Lee‐Woolf (2009) mentions that the food chain generates large food waste and requires unsustainable water usage. However, Lee‐Woolf (2009) advocates for community‐based approaches, explaining that they are an integral component of establishing a sustainable food system in the UK. Modern literature has also discussed the UK food system's unsustainable practices, policies and principles. For example, Ghadge et al. (2020) believe that implementing sustainable practices in supply chains will help overcome existing and emerging challenges. They analysed the UK's artisan cheese supply chain and found internal and external barriers preventing sustainability. For instance, producers are concerned that transitioning to sustainable practices will increase the cost of artisan cheese, already consumers pay specialist prices compared with regular cheese (Ghadge et al., 2020).

The UK's decision to disassociate itself from its geopolitical commitments as part of the EU opens more opportunities for its dairy industry and agricultural sector to be reshaped and entirely self‐regulated (Holmes, 2018). Holmes (2018) suggests that transitioning from raising livestock to pulses dominant agricultural sector would aid the UK in attaining some of the SDGs environmental targets, that is reducing emission and boosting biodiversity. Whilst the external barriers are less controllable because international and European governing bodies frequently implement new environmental legislation in the dairy industry (Ghadge et al., 2020), Brexit could be the drastic change necessary for helping the country's food system progress towards sustainability (Holmes, 2018).

##### External factors

As mentioned above, external factors impact and dictate some of the actions and practices observed in the UK's food supply chains. For instance, Tranter et al. (2007) highlight the significant impact of EU policies on food production, land use and rural development in Britain. The single farm payment (SFP) was part of the attempt to reform the EU common agricultural policy (CAP), where subsidy payments were decoupled from production expectations (Tranter et al., 2007). Farmers were able to cease production entirely and prioritise other ventures; however, to qualify for this payment, certain conditions had to be met, that is animal welfare and environmental requirements, good farming practice (Tranter et al., 2007). Various concerns were raised in the paper as it was believed that the scheme would cause the abandonment of farms as farmers opt out of conventional agriculture activities and responsibilities (Tranter et al., 2007).

Furthermore, this led to the rationale that idle farmland would reduce the national food supply. Tranter et al. (2007) research was based on the potential impacts of the SFP; hence, a lot of the impacts were based off assumptions; thus, follow‐up research is necessary to quantify and discuss the observed impacts of the policy. Olagunju et al. (2020) suggest that the decoupled payments have negatively impacted production levels in Northern Ireland (NI) as the range of production levels in the country widens. As these observations and calculations have been made on a small‐scale, the full‐scale impact of the SFP remains unclear. Regardless, Olagunju et al. (2020) reveals the potential influence of decisions made by external governing bodies in altering farmers' attitudes and agricultural cultivation levels. This rationale is further demonstrated by Ridler (2017), who believes that pesticide regulations enforced by the EU threaten the future food supply of the UK. Ridler (2017) elaborates by explaining that the ban of herbicides containing glyphosate will limit the protection tools available for agriculturalists, thus increasing the likelihood of crops developing resistance. The governmental support for implementing this policy is perceived to be driven by underlying political motivations rather than an attempt to enforce sustainable cultivating practices (Ridler, 2017). Due to Brexit, it is unlikely that future EU's decisions relating to the use of specific types of pesticides may impact the UK. Whilst this policy may no longer pose a threat to the UK's agricultural sector, newer policies such as the Northern Ireland Protocol as part of the UK's withdrawal agreement from the EU may hinder trading between the UK and remaining EU member states (BBC News, 2022). The protocol requires the EU member states to adhere to strict health and safety precautions on imported food from non‐EU countries. Consequently, many British food businesses have restructured their supply chains as they decide to stop supplying to Nothern Ireland (Speciality food, 2021).

This further demonstrates external governing bodies' sheer influence on dictating domestic and international legislation, regulations and practices. Ridler's (2017) blog is essential as it brings minority and polarised views of agriculturalists and small food businesses to the mainstream, by discussing potential concerns for those within this community.

Similarly, O'Carroll (2019) airs out farmers' grievances regarding the detrimental impacts of potentially having a ‘no deal Brexit’ on the UK's food supply. O'Carroll (2019) explains that by leaving the EU, the British government will be directly responsible for ensuring the country's food supply and food safety. This reinforces the idea that even though leaving the EU would increase farmers agency, there are different challenges which are associated with transitioning towards sustainability and national food security. Concerns surround whether decision‐makers will establish the necessary support needed for the industry and its stakeholders, especially small farm holders, to thrive despite this transition (O'Carroll, 2019).

##### Small and local food chains

When conceptualising the food system, the role of local food systems in attaining food security is often overlooked (Maye and Kirwan, 2013). Typically, food security is perceived as a global issue that is addressed using sustainable intensification, market liberalisation and risk management (Maye and Kirwan, 2013); therefore, small‐scale approaches are not frequently utilised. Maye and Kirwan (2013) argue that local food chains are significant responses to acute food insecurity. They use the example of the ‘Dig for Victory’ campaign nationally promoted in the UK in 1939 to resolve the food shortage which resulted from the German U‐Boat blockage. The campaign encouraged individuals, families and households to utilise their allotments and open public spaces, that is municipal parks, to alleviate the pressure on the agricultural sector by cultivating their own produce. However, Maye and Kirwan (2013) recognise that for the significance of local food chains to be acknowledged, it requires the reframing of the concept of food security. By recognising that food insecurity is a multiscalar issue, it allows for establishing approaches and measures on different scales.

Oglethorpe and Heron (2013) discuss the constraints in the UK local food supply chain; they mention that one of the significant barriers is that local food markets are located rurally, therefore excluding urban consumers. They suggest that urban consumers may better access local food through multiple retailers; however, as already recognised by Hingley et al. (2016), the relationship between small suppliers and large food retailers and buyers is complex. Thus, small producers have had unsavoury experiences when exposed to large organisations and believe that the collaboration would not be financially and operationally viable (Hingley et al., 2016). The decision not to sell to large retailers continues to stunt the growth and performance of local suppliers as they are unable to fulfil urban demands. Despite this, participants in Schmutz et al. (2017) interview identify that small FSCs perform better than mainstream methods of food supply as they are human centred. Equally, Schmutz et al. (2017) found that community‐supported agriculture delivers the highest overall social, economic and environmental benefits. Respective of the optimistic findings regarding the sustainability and importance of small and local supply chains, there needs to be more research on how to create smaller food supply chains in urban contexts. Freedman et al. (2016) argue that farmers markets help to provide a level of access to shorter FSCs within cities. But more research is needed to understand how to better encourage and support the development of smaller supply chains in the system especially in urban settings.

##### Lack of research and development

Kirwan (2004) similarly discusses farmers markets as possible alternatives within the UK food system, focussing on the engagement between producers and consumers. Kirwan (2004) concluded that further research is needed to fully comprehend the driving motivations for producers and consumers' involvement in food markets. Kirwan (2004) argues that understanding consumers is particularly essential as they have become more reflective and conscious of their food choice; hence, their food purchases are influenced by their beliefs. Lastly, Kirwan (2004) reinforces the importance of having alternative strategies such as farmers markets as they help reconnect the cultural, social and environmental context of food production. However, Kirwan (2004) recognises that further research is necessary to sustain these strategies in the UK's food system. Although the article was informative in helping the reader realise the importance of research and development, there were no recommendations as to how to practically apply these findings. Thus, it is unclear as to what type of support would be required from the government for these alternative schemes.

In contrast, Ingram et al. (2013) identified and outlined priority research questions relating to the UK food system from participants made up of different stakeholder groups, that is the private sector, NGOs, advocacy groups, policy and academia. The questions ranged across 10 significant themes such as production and government policy (Ingram et al., 2013). They explain that the wide range of questions reflects individual stakeholders' realisation that more research needs to be conducted on various aspects of the food system (Ingram et al., 2013). Currently, public finance is focussed on food production research as this is perceived to be a dominant method of attaining national food security agendas (Ingram et al., 2013). Thus, they highlight the importance of integrating research on food system activities to food security outcomes alongside increasing the collaboration between academics and practitioners in research projects. Doing this will help to consider the different perspectives and priorities of stakeholders when suggesting solutions (Ingram et al., 2013). The list of priority questions has highlighted opportunities for cross‐sectional partnerships between food system disciplines (Ingram et al., 2013). However, the use of a systems approach has provided a broad range of questions relating to the UK; thus, it sacrifices giving readers a deeper insight into why specific questions are being raised by stakeholders. Moreover, although systems thinking provides a balanced discussion across all areas and activities in the food system, using this approach has not effectively narrowed down the areas for the government or practitioners to focus on. Rather Ingram et al. (2013) findings provide a list of questions suggesting that all areas of the food system should be prioritised. This is an impractical and inefficient way to increase research and development of FSCs as it does not provide a clear direction; this may lead to delays in decision‐making. Also, a lack of direction leaves stakeholders to decide which areas of the food system are most significant to them, where sustainability may not be prioritised.

#### 3.1.2 COVID‐19 pandemic period

##### Social, economic and environmental impacts

The pandemic has had social, economic and environmental consequences for society as supply chains are disrupted. For instance, the period magnified the social injustice across the food industry, Henderson (2021) discusses the poor treatment of horticultural workers. The virus worsened the exploitation of seasonal migrant workers, as whilst everyone else was shielding and self‐isolating, they continued to meet consumer demands (Henderson, 2021). Similarly, migrants are economically exploited as laws and regulations do not protect them (Henderson, 2021). Low wages have made it difficult for migrants to travel back home during the pandemic. Additional costs associated with travel restrictions, that is covid tests, increased flight prices and quarantining were unaffordable (O'Connor & Evans, 2021). The pandemic highlighted the UK's food system's reliance on migrant workers. This reinforces the rationale that the UK needs to establish FSCs centralising domestic labour (Henderson, 2021). Such discussion is increasingly relevant as Brexit legislations start to take effect—restrictions on travel for seasonal migrant workers entering the country. Barling (2020) argues that the UK must improve working conditions by increasing rights and re‐evaluating immigration laws to construct better social and economic environments for seasonal agricultural and horticultural workers.

Likewise, Harvey (2020) discussed the impact of the pandemic on agricultural labour; however, Harvey (2020) also considers the risk posed to farmers during this period. Harvey (2020) explains that the average age of a UK farmer is 59; hence, many farmers are especially vulnerable to COVID‐19. As fewer young people launch careers in agriculture, societal perceptions and attitudes towards farm work are examined. The lack of appreciation for farmers has been exacerbated during the pandemic. Their life‐risking efforts to provide food for the nation went amiss amongst the public who only expressed their gratitude for NHS heroes. Moreover, the Daily Mail's publication of a political advisor's opinion that ‘Britain does not need farmers' has further reinforced the perceived insignificance of agriculturalists (Owen, 2020). Thus, strengthening the food system would require the social reframing of agricultural work, resulting in an increased uptake of careers in agriculture amongst younger generations.

Unlike the social consequences, the environmental impacts of modern food supply chains have been more overtly acknowledged. Aikins and Ramanathan (2020) identify the various aspects of the UK FSC that emit the most carbon. They found that transportation and distribution are the two main emitters of carbon as UK supply chains span across multiple nations (Aikins & Ramanathan, 2020). Importation is one of the country's leading methods of acquiring food (Ingram et al., 2020). Moreover, the article mentions that there have been various attempts to reduce the carbon emissions produced in the UK food system through collaborative efforts, advocating the use of local chains and carbon labelling (Aikins & Ramanathan, 2020). However, some of these methods have been insufficient in reducing energy consumption and carbon footprint. This suggests that policy‐oriented approaches may be a better method of reducing carbon emissions (Aikins & Ramanathan, 2020). Aikins and Ramanathan (2020) conducted a multilinear regression (MLR) on secondary data; thus, their discussion was based on the numerical outcome. However, Almalki (2016) argues that quantitative or qualitative methods alone may produce results that do not holistically reflect the depth and complexity of problems. Aikins and Ramanathan's (2020) research was published during the pandemic; however, the secondary data used for the MLR were collected from 1990 to 2014. The findings do not accurately reflect carbon levels before or during the pandemic. However, Cummins et al. (2020) insinuate that the COVID‐19 pandemic has had environmental benefits, as due to restrictions consumers are utilising their local food environments more; therefore, it can be inferred that carbon emission may have also reduced during this period.

COVID‐19 restrictions also had economic impacts. The pandemic caused more consumers to source their produce from local food retailers and use digital delivery services. This facilitated the relocalisation of the urban food retail system (Cummins et al., 2020). Despite this, there is growing concern that many independent local food businesses and retailers may not survive the postpandemic recession as consumers resume conventional food sourcing practices, that is purchasing from mainstream supermarkets and retailers (Cummins et al., 2020). Moreover, a weaker economy postpandemic could result in lower overall takeaway and restaurant sales as fewer people use these services and instead prepare food from home (Cummins et al., 2020).

The pandemic impacted independent restaurants and businesses more than chain restaurants. To mitigate the economic downturn post‐COVID‐19, Cummins et al. (2020) emphasises that long‐term financial support from the government is necessary. However, they did not indicate what this financial support would look like or how best to provide this support, that is government schemes; hence, this creates a level of precarity for small local food businesses.

##### Systematic failures

Systematic failures such as a lack of resilience, uncertainty and vulnerability were frequently acknowledged and discussed in relation to the UK food system. Publications explained why these flaws posed a serious threat to the longevity and efficiency of the country's food production system. For instance, Moran et al. (2020) argue that aspects of the UK FSCs lack resilience. Conversely, they mention that the pandemic revealed that the country has significant adaptive capacity. This was demonstrated by how retailers could rapidly meet increased demands (Moran et al., 2020). In contrast, Mitchell et al. (2020) recognise that despite the significant disruption caused by the virus, new FSCs demonstrated a high degree of resilience. Do et al. (2021) agree with this finding. They explain that during the pandemic, impacted supply chains, such as bread, were able to cope despite increased demand. However, Mitchell et al. (2020) mention that the food system lacks adaptive power as it has shown signs of being stuck in a ‘rigidity trap’. They explain a system centralising large‐scale producers and suppliers creates inflexibility in the UK production system, thus increasing overall vulnerability to shocks (Mitchell et al., 2020).

The conflicting findings of Moran et al. (2020) and Mitchell et al. (2020) reflect the differing perspectives of the early stages of the pandemic. As both literature used empirical research as the methodology of their study, often this method is critiqued as being overly subjective; hence, it can be assumed that to an extent each of their findings was shaped by unconscious bias (Mitchell et al., 2020; Moran et al., 2020). Factors such as authors' proximity to the problem, location (i.e. urban or rural), individual experiences and level of knowledge about the UK food system may have contributed to these differences. Moreover, the paradox of Moran et al. (2020) and Mitchell et al.’s (2020) findings can also be explained by the fact that the former focuses on the general resilience of the UK food system, whereas the latter investigates fresh food supply chains. Therefore, both findings can be true in the sense that on a micro‐scale, some supply chains in the UK food system are resilient during the pandemic, whereas on a macro‐scale, the system is not resilient. Irrespective of these juxtaposing views, both literature carries a hopeful tone, which suggests that the authors are optimistic that these systematic flaws can be resolved (Moran et al., 2020), with investments in innovative solutions and R&D (Mitchell et al., 2020). Systematic failures have similarly been discussed in the literature (Elliott & Bhunnoo, 2021). While these are written in the later periods of the pandemic, they prioritise detailing methods and approaches for postpandemic recovery. For instance, Elliott and Bhunnoo (2021) outlines and discuss scenarios for transforming the UK food system to align more with global targets, that is the Paris Agreement and the Sustainable Development Goals (SDGs).

##### Food waste

Reducing food waste is among the targets for goal 12 of the SDGs (Thapa Karki et al., 2021). Goal 12 focuses on encouraging responsible consumption and production. It aims to halve global waste at all levels of the food production system by 2030. Similarly, Cao et al. (2020) recognise that waste occurs at all scales, from producer to household but especially at the manufacturing stage. Consequently, they have established a method to reduce edible food waste. They explain that waste results from internal and external factors and addressing this issue effectively requires a collaborative effort by stakeholders on various scales (Cao et al., 2020). In comparison, Thapa Karki et al. (2021) found a coordinated effort between multiple actors in the chain at the city level; however, there are tensions and challenges despite this. They explain that as the flow of food goes from commercial to noncommercial supply chains, a linguistic shift occurs as ‘food waste’ becomes ‘surplus food’ (Thapa Karki et al., 2021). The differing perspective and attitudes towards food waste/surplus food reflect why different food organisations take different approaches to managing waste. Food alliances play a significant role in ensuring the prioritisation of reducing food waste by preventing logistic inefficiencies and creating cohesive food supply chains as players within the system may prioritise fulfilling independent goals, that is profit‐making (Thapa Karki et al., 2021). Both literature acknowledge that food waste is an inevitable by‐product of the food production process; however, the focus continues to be on understanding the most effective methods of minimising waste and how to best utilise the waste produced (Cao et al., 2020; Thapa Karki et al., 2021). However, neither of the literature acknowledges consumers' role in contributing to food waste production, thus placing the onus on other stakeholders, consequently diminishing any responsibility consumers have in waste management. Patel et al. (2021) emphasise the importance of comprehending consumers' decisions, attitudes and behaviour towards food as they have found that the highest level of avoidable food waste is produced at the household level.

##### Consumers' decisions and behaviour

Consumers' attitude and behaviour have increasingly become an area of concern, as Draper et al. (2019) explains that consumers do not care about where their food comes from; they were more concerned with the flavour and the taste. Furthermore, they found that proximity to food supply chains also impacted the level of knowledge consumers had; those living closer to FSCs were more knowledgeable than those living further away (Draper et al., 2019). However, Draper et al.’s (2019) interviewed participants who voiced their fears about being exploited by suppliers and retailers as they believed that not enough regulations were in place to prevent such exploitation. Barling (2020) discusses how the government continued to protect the interest of consumers during the pandemic by briefly waiving the Competition Act, which ensured food security for consumers. This further reinforced Draper et al. (2019) finding that consumers lacked knowledge about how the government protect consumers using regulations. In contrast, Draper et al. (2019) argue that consumers' ignorance is at times strategic as this allows them to continue their mundane routine as well as a method of showing their trust in the system and regulations in place. However, COVID‐19 has increased the visibility of the previously covert issues in the UK FSC, resulting in consumers' distrust in the food system reflected in behaviours such as stockpiling.

Similarly, Armstrong and Reynolds (2020) mention that consumers have a ‘folk’ level of understanding; hence, they are reliant on labels as a form of acquiring knowledge surrounding food. They explain that the consumers' limited knowledge fosters buying habits that reflect consumers' scepticism about where specific foods are produced. For example, they found that foods produced in China and the United States were typically viewed in a less favourable light than Fairtrade and organic produce alongside food cultivated in Europe and the UK (Armstrong & Reynolds, 2020). Draper et al. (2019) explain that consumers are less suspicious of locally sourced and produced food as they believe the EU and the UK have the best food quality regulations. Berry and Romero (2021) support the findings of Armstrong and Reynolds (2020) as they explain that some consumers use package claims and labelling to gain more knowledge of the product in which they then base related or unrelated inferences.

Consumers' limited knowledge of the UK food system is a concern as it could also reflect their lack of acknowledgement of the significance of their role as perpetrators or as potential solutions to some of the existing challenges in the UK food system, that is food waste. Moreover, as the UK engages in trade with countries outside the EU because of Brexit, it will increase the opportunity for food fraud creating a less secure food environment for consumers (Brooks et al., 2021). Ensuring food safety is one of the many challenges facing the UK food system. Thus, consumers must understand how social, economic and political changes can directly impact the quality and access to food. Therefore, it is beneficial for consumers to engage in food dialogues and discussions at least at an intermediate level.

#### 3.1.3 Re‐occurring challenges

Amongst the different publications, the most significant challenge identified was food insecurity, as it was recurringly discussed as a growing concern for the UK before and throughout the pandemic (see Table 3). Since 2010, food security has increasingly been explored. Early literature such as Barling et al. (2010) focussed on outlining past food policies and discussing their shortfall concerning attaining food security in the UK. They highlighted that Defra believed that developed countries such as the UK do not struggle with food insecurity (Barling et al., 2010). These issues were associated with poverty; therefore, food insecurity was perceived to be a distant reality for the country. The UK's policies aimed to prevent food insecurity rather than resolve it as economic approaches were used to increase food supply by importing more food to ensure consumers' demands were met (Barling et al., 2010). Lee and Marsden (2011) recognised that these practices were not sustainable as they considered future food scenarios and found that the country's food security is vulnerable to changing global market conditions as pressure on supply would result in inflated prices.

**TABLE 3:** Food security/insecurity

References	Literature type	Research aims	Method	Main findings	Practical applications
Barling et al. (2010)	Book Chapter	To outline and describe the different policies implemented to facilitate national food security. To outline and describe the strategic policy agenda to attain food security in the UK. Identify the shortfalls of past policy approaches to addressing food insecurity.	Qualitative/Secondary Research	Importing food was used to increase food security as it was perceived to increase resilience and flexibility within the food system. Policies took an economical approach to understanding and addressing food concerns. Defra believes that developed countries like the UK do not struggle with national food insecurity. Food sufficiency and food security were two conflicting ideas in food policy.	To highlight some of the sustainability challenges regarding food security in the UK. To outline some of the progressions which have occurred in food policy. To highlight areas for improvement and further research, i.e. environmental constraints being fully embraced in the UK government's food supply and security policy statements. To raise awareness of some of the emerging political discourse around the UK food security.
Lee and Marsden (2011)	Journal article	To demonstrate how future food scenarios might be analysed from a systems' transition perspective to understand what is needed to transition towards a more sustainable and resilient food system (production and consumption). Gathering empirical data using food futures scenarios.	Qualitative/Secondary Research	Based on future food scenarios, changing global circumstances would result in pressure on supply, resulting in inflated food prices. A transition to a more sustainable system is necessary; however, there are doubts about feasibility as the UK agricultural capabilities are reduced. Research and development are essential to increase sustainability and technologies to improve production. Good leadership and governance are also crucial.	To reinforce the importance of developing sustainable and resilient food systems. Raise awareness of some of the potential social and economic challenges if more effort is not made to increase the sustainability of systems. Challenging conventional perceptions that the UK food system is sustainable.
Tomlinson (2013)	Journal article	To give an analysis of the claim that we need to increase global food production to feed the world in 2050. To show how statistics are critical discursive devices used by institutions and individuals to promote and push the ideological framing of food security issue.	Qualitative/Secondary Research	‘Wrong’ statistic has played an essential role in pushing dominant discursive framing of food security in the UK. A focus on statistics presents food insecurity as a lack of food supply and overlooks other causes. Increasing food supply is problematic as it does not address climate change, diet‐related health problems nor substantially reduce absolute hunger.	To challenge the dominant framing of the problem of food security in the UK and its resolution. Raises awareness of the potential detrimental impacts of using statistics as a tool to explain food insecurity as it paints an inaccurate narrative. Food insecurity is complex and not solely due to a lack of food supply.
Tsolakis and Srai (2017)	Journal article	To investigate the role of smallholder farming in tackling food security and sustainability‐related challenges in the developed world. To develop a system dynamics modelling framework that captures the self‐sufficiency of cereal production in the UK.	Qualitative/Secondary Research and Simulations	Implementing a policy intervention to develop new Small Farms (SFs) could prolong the UK food security state and increase the region's average annual gross domestic product. SFs consume less fertiliser than existing farming systems on an annual average, thus having reduced environmental impact. The development of SFs provides employment opportunities and public health implications.	To advocate for the development of small farms by helping decision‐makers realise the benefits of small farmers. To highlight that food security in developed countries is a multidimensional and complex issue, thus requires a combination of approaches to resolving the issue. To motivate focussed research on robust policy‐making interventions that promote small farms as sustainable short food supply chains.
Price (2020)	Blog	To inform readers on how the UK's food system is being impacted in the early stages of the COVID‐19 pandemic.	Qualitative/Secondary Research	Food insecurity increased in the UK during the pandemic due to restricted access, especially amongst vulnerable groups, that is older adults. More people became reliant on food banks in the early pandemic stages; food banks were under pressure as there were fewer donations during this period. Just‐in‐time food supply chains have been one of the leading causes of food insecurity during the pandemic.	To inform people of the various challenges during the pandemic relating to food in the UK. Reinforce the importance of the government and institutionals role in supporting and mitigating the impact of the pandemic on disadvantaged groups' access to food.
Power et al. (2020)	Journal article	To outline the main challenges existing in the UK food system and recommend solutions and methods for improvement.	Qualitative/Secondary Research	Just‐in‐time supply chains have been challenged by stockpiling behaviour contributing to the food system's vulnerability as a whole. The food aid system is unable to withstand the sudden high demands during health and economic emergency. The government are responsible for protecting population health, ensuring household incomes and safeguarding the economy.	To help end the 5‐week wait for Universal Credit and advocate for cash grants for low‐income households. To help the central and local government recognise that many food aid providers are already at capacity and cannot take on additional responsibilities. Thus, other forms of support need to be established. To help ensure that adequate policies which address the social and economic issues surrounding food are established. To suggest new policies or ways to improve existing policies to reduce food insecurity.
Bhunnoo and Poppy (2020)	Journal article	To outline a programme for transformation of the UK's food system, for healthy people and a healthy environment. To identify some of the social, environmental, and political food system challenges in the UK.	Qualitative/Secondary Research	The article recognised that there are challenges to national policy‐making in the context of food security. A better integrated approach is necessary for establishing food policy and resolving food insecurity. Integration is difficult to achieve as it would require a new approach to governance.	To educate the audience and raise awareness of some of the challenges acknowledged in the UK food system. Identifies areas for improvement within the system, mainly focussing on policy. Provides suggestions to improve current policies and practices. To promote the development and implementation of sustainable and effective policies relating to food.
Karki, Bennett and Mishra (2021)	Journal Article	To examine the distribution supply chain of surplus food distribution at the city level.	Qualitative/semi‐structured interviews and participant observation	There is a need for a coordinated effort between actors to help capture the value of surplus food. There is a hierarchical power relationship between organisations in the supply chain. There is a lack of a legislative framework for food donations which poses other challenges for third‐sector organisations.	Emphasising the importance of establishing a legislative framework to regulate the donation of surplus food from supermarkets. Raising awareness of areas for future research relating to finding practical solutions for managing waste and surplus food, that is using digital platforms.

Furthermore, Tomlinson (2013) adds that a focus on increasing food supply is problematic as it does not protect against climate change, diet‐related health problems and does not considerably reduce hunger. Thus, this reinforced Lee and Marsden's (2011) belief that a transition towards sustainability is necessary to reduce vulnerability and increase the resilience of the country's food system. Although they acknowledge that the UK's agricultural capabilities are limited, they were confident that the transition to sustainability is feasible with investment in R&D, technology alongside the right systemic support and governance (Lee & Marsden, 2011). Reframing how food security is conceptualised was a vital critique of the early 21st century approaches as Tomlinson (2013) explains that statistics were used as devices by institutions to perpetuate ideological framing of food security in the UK. Despite the establishment of the SDGs in 2015, which reinforced that hunger caused by food insecurity is a global issue, the narrative that food insecurity was not a concern for the UK and developed countries alike continued to be perpetuated (Pollard & Booth, 2019). This misconception led to governments retreating from related issues and left third‐sector organisations with the responsibility of rendering services to those in need (Pollard & Booth, 2019).

Pollard and Booth (2019) explains that the definition of food insecurity shapes the response given; they argue that societal benefits and food waste mitigation are the two dominant framings of food insecurity in developed countries. The government's response has mostly been through food assistance, which does little to resolve the underlying causes of food insecurity (Pollard & Booth, 2019).

Similarly, during the pandemic, more publications identified that the UK grapples with food insecurity. Lasko‐Skinner and Sweetland (2021) recognise that food insecurity levels in the UK are among the highest in Europe and have been exacerbated by the pandemic. Unlike earlier approaches, many publications are investigating the underlying social, economic and political causes of food insecurity and discuss methods to tackle it. For instance, Tsolakis and Srai (2017) believe that one of the leading causes of food insecurity is the unsustainability of the UK food system, suggesting that decentralising the country's food supply chains by supporting the development of smaller farms would help progress towards sustainability. Also, they mention social, economic and environmental benefits of having small farms, that is reduced fertiliser consumption, increased employment opportunities and average annual gross domestic product. Price (2020) and Power et al. (2020) agree with Tsolakis and Srai (2017) observations that the modern food system is unsustainable and demonstrate this rationale by discussing the consequences of relying on just‐in‐time (JIT) supply chains during the early period of the pandemic. The JIT chains were unreliable and inefficient in meeting the needs of consumers because demands increased significantly due to stockpiling, but the food was not readily available at the same rate (Power et al., 2020).

Consequently, this led to increased food insecurity amongst vulnerable and disadvantaged groups in society, that is the elderly persons and individuals from low economic backgrounds (Price, 2020). Another reason food insecurity has become more pertinent and visible in UK communities, especially during the pandemic, because food aid systems were unable to act as an effective secondary source of access to food (Power et al., 2020). Third‐sector food organisations rely on donations; however, there were minimal donations during this period (Price, 2020). Thapa Karki et al. (2021) believe this is due to the lack of legislative framework and government support for third‐sector organisations, as no one is accountable for ensuring that non‐profit organisations can adapt and cope with sudden changes in demand. Similarly, Bhunnoo and Poppy (2020) recognise difficulties in national policy‐making regarding food security. They suggest that more collaborative approaches are necessary for developing policies to resolve food insecurity by integrating different sections of the government and facilitating cross private sector engagement (Bhunnoo & Poppy, 2020). However, the critiques and discussions had in food insecurity literature (see Table 3) are also relevant and give an insight into the ongoing health crisis and UK's attempt to tackle and mitigate the impacts. However, as Power et al. (2020) and Price's (2020) work were written in the early periods of the pandemic, there was insufficient evidence at that time to show how the pandemic was impacting food insecurity in the country. Their discussions were formulated in the context of previous literature on these topics. Irrespective of the contributions of Power et al. (2020) and Price's (2020) literature are vital for ensuring food insecurity remains at the forefront of government priority throughout the pandemic. Although there have been clear progressions mainly in the efforts to reframe conventional assumptions of food security, the health crisis has worsened existing social and economic conditions, compounded with Brexit adding to the complexity of resolving food insecurity in the UK.

## 4 DISCUSSION

### 4.1 Limitations of this comprehensive review

Although the inclusion criteria was used to achieve neutrality, subjectivity is inevitable during the screening process; research bias cannot be eliminated entirely. Researcher bias occurs as it is the researcher's decision on which literature to include based on how they have interpreted the inclusion criteria; also, they decide the depth in which different papers are discussed. For instance, the decision to include grey literature in this review may increase subjectivity and reduce the validity of the findings as some publications are based on perspectives and have not been peer‐reviewed. However, the fixation on achieving internal validity results in a skewed portrayal of reality; thus, these limitations may undermine the suggestions, claims and findings of the comprehensive reviews as a standardised research method. Before conducting this research, these limitations were considered, and it was concluded that the strengths of using a comprehensive review outweigh the weaknesses.

Moreover, there are limitations with particularly focussing on the literature published during the pandemic as its impact has varied in intensity due to the response used at different stages of the spread of the virus. Thus, the literature will reflect the stage of the pandemic in which it was written. It is difficult to compare the literature in that regard, rather they will be used to create a timeline outlining the various challenges which occurred and may have worsened due to the health crisis. Also, as the pandemic is progressive, the literature has not been able to fully quantify the effects of the virus on the UK's food system; hence, some of the publications may make assumptions about the possible medium to long‐term impact of COVID‐19. Another limitation with focussing on literatures published during the 2019–2021 is that it perpetuates the thinking that food issues are only a concern, specifically in developed countries, during sudden outbreaks which disrupt quotidian social, environmental and economic conditions.

### 4.2 Practical takeaways

This review reveals that food insecurity is a leading problem in the UK food system. More importantly, the review reinforces Horner's (2019) rationale no country's development approach is superior to another. Horner (2019) explains that across different dimensions of development, places and people in the developed and developing world have been observed as facing many shared challenges, as some underdeveloped parts of the Global North bear a solid likeness to parts in the Global South (ohchr.org, 2017).

Undeniably, the UK is more progressive socially, economically and politically in comparison with other countries. Yet, the UK must acknowledge that there are fundamental aspects of its overall system that are in dire need of transformation. The UK food system is underdeveloped not in the conventional way in which people may typically comprehend or envision underdevelopment, as the country possess the financial resources and frameworks to support the necessary changes. But the country has become accustomed to its codependent relationship with the EU as it provided support and guidance through regulations and trade. However, as the UK continues on its journey to detach from the influence of the EU, the challenges within its food system will become more pronounced.

#### 4.2.1 Mitigating the impact of Brexit on stakeholders

Despite the impacts of the UK leaving the EU are widespread, the most pertinent impacts are reflected in sectoral regulations, governance and trade. These progressions have caused multiple changes in the UK food sector; hence, many stakeholders have lacked the necessary support needed to understand their current roles and responsibilities within the new systems.

Impacts of Brexit are inevitable as developing a sustainable national food system independently from the EU, which adequately meets the demands of 21st century consumers, is challenging. As there are various components of the UK food system which have to be reformed, reworked and restructured. Therefore, the government will not be able to completely shield stakeholders from the implications associated with Brexit; however, they can mitigate the effects. There are different ways in which the government can mitigate the impacts of Brexit on different stakeholders in the UK food industry.

##### Producers

Defra is piloting environmental land management schemes (ELMs). These schemes support farmers by paying them for taking on increased roles and responsibilities as environmentalists through sustainable farming actions, among other things (Sustain, 2022). The schemes will be tested over the next 3 years to gather knowledge on the success and pitfalls of the schemes and how well they interact with one another. This will help bolster farmers' overall income as they will be paid for their farm's production as well as their efforts towards achieving sustainability. However, there are ways in which Defra can improve the dissemination of these schemes. For example, the combination of complex schemes and a lack of communication have left farmers confused and concerned about how the different schemes will fit together. Therefore, increasing stakeholder communication is a simple way to defuse some of the scepticism surrounding ELMs and provide clarity (Sustain, 2022). Furthermore, providing farmers access to advisory services will help support farmers in making the best decisions regarding which schemes they are best suited to and how best to maximise their financial capabilities (Sustain, 2022). This particular support is essential for small farmers as they have to compete with large supermarkets as well as try to adjust to sectoral changes due to Brexit. Such improvements provide producers with the necessary support required to comfortably survive and thrive during this transition period.

##### Businesses

The provision of clear advice and documentation better prepares businesses for changes to trading regulations – such as the EU‐UK Trade and Cooperation Agreement (TCA) (Dentons.com, 2020). Without this, businesses may fall victim to having to pay tariffs or fines if they fail to meet the criteria set in the agreement. In addition to this, the government can minimise the impact of Brexit on the country's food businesses by forming relationships and attractive agreements with other countries outside of the EU. This will help to create new trading opportunities for businesses which would help to mitigate potential increases in transportation costs due to EU tariffs.

##### Consumers

To protect consumers against the impacts of Brexit, the government should prioritise ensuring that economic and social conditions are as stable as possible. The increased cost of living as a result of Brexit‐induced increases in the cost of food production – disruptions in transportation, new tariffs and currency volatility – are passed on to consumers. These lead to increased food insecurity (Holland, 2022). Therefore, the government should provide financial support to ensure people can afford necessities such as food or ensure that the cost of other core living expenses, that is housing and important resources such as fuel, remain affordable to allow people to be able to spend more on food.

Secondly, changes in food standards have caused concern among consumers as these were major responsibilities held by the EU. However, the government can initially retain food standards from the EU before transitioning to new regulations (Lerigo, 2020). This reassures the public that new standards are well‐considered, and their food will remain of high quality. This would allow consumers to carry on as usual without having to be concerned about the quality of the food they are consuming (Lerigo, 2020).

As long as the government provides tailored support to different stakeholders in the food sector, there are multiple ways in which the government can help mitigate the impact of Brexit beyond the methods described in this section.

#### 4.2.2 Improving the UK food system

Brexit is the UK's opportunity to start prioritising the development of its food industry. The country's first action would be to reframe its mindset towards domestic food production. A Eurocentric mindset provides a level of privilege of not having to care about resolving core domestic issues such as food insecurity as they can rely on global imports to sustain the country. Whereas, developing countries do not have the financial means and trade partnerships to afford such a luxury. This lackadaisical attitude has contributed to the rising cost of living in the UK. One in five of the UK population (22%) are in poverty—14.5 million people (Joseph Rowntree Foundation, 2022); with the poorest tenth of households spending proportionally higher on food and fuel compared with the richest (Stewart, 2022). Notably, food insecurity is the visible outcome of the compounding of several issues within the country's food system. The government's current approach portrays the growing food insecurity in the country as a surface‐level issue, as they direct their efforts on increasing public access to nutritional food rather than simultaneously addressing core problems in the industry. Similarly, the Queen's 2022 speech's failure to mention actions to increase food security reinforces the government's flippant attitude to developing a sustainable food system. If the UK were to carry on in this trajectory, it threatens its development status as more of its citizens falls into deprivation (Sandercock, 2022). With the increasing cost of living, it has never been more essential to restructure the UK's food system by ensuring policies and practices provide sustainable solutions to tackle food insecurity. As well as stepping away from its Eurocentric lens and increasing self‐sufficiency.

The rest of Section ‘Practical takeaways’ provides a brief description outlining social and political changes the UK government can adopt to improve the condition of its food sector.

#### 4.2.3 Restructuring and regulation

**Challenge:** Lack of support for British farmers

**Solution:** The Agricultural Transition Plan (ATP) 2021 to 2024 set out by Defra has reinforced the additional pressure on agriculturalists to be drivers of sustainability as well as being responsible for national food production. The Transition plan focuses on making farming more lucrative assuming that through this type of support farmers will be more inclined to produce food. However, equal emphasis needs to be placed on production and ensuring farmers have the right support and are equipped with the necessary knowledge on how to make their practice more sustainable. This will require collaborative efforts from academics and sustainability experts in working with farmers to ensure that they can develop sustainable practices in conjunction with maintaining and in some instances increasing productivity. de Boon et al. (2022) work demonstrate stakeholders' concern regarding the ATP's focus on addressing climate change and biodiversity loss and the lack of prioritisation of the production of food and fuel. Through collaboration, farmers will receive more support which will allow them to direct more of their efforts to production. This is a micro‐scale solution; however, a more macro‐scale solution would be to ensure clear, consistent policies and regulations are established across the entire industry. This would guide farmers as currently, there are concerns regarding the lack of clarity of Environmental Land Management schemes (ELMs) as it provides little detail as to how the new support schemes will work (Marshall et al., 2022).

#### 4.2.4 Merging rural and urban environments and practices

**Challenge:** Low food production in Urban cities

**Solutions:** High populations in urban cities have increased the demand for food, housing, jobs and resources. During the pandemic, food transportation methods were disrupted; hence, it reinforced the importance of developing shorter food supply chains by increasing food production in urban environments. Urban horticulture is imperative for combatting food insecurity, developing resilient regional food systems, and addressing global sustainability issues. This can be achieved through establishing vertical farms, rooftop agriculture and farmers markets as well as increasing community gardens and allotment spaces in cities. During the World War II, Britain witnessed the value of allotments and community gardens in mitigating the impacts of food shortage, similarly during the pandemic, allotment renters were amongst those consistently replenishing food banks with fresh fruit and vegetables (Barrie, 2020). Relocalisation of food production is necessary for progress towards food security and sustainable food systems (Lopes, 2021); as allotments, community gardens, and farmers markets benefit the local communities and urban farmers (Willis, 2012).

#### 4.2.5 Increasing interest in agriculture

**Challenge:** Stigma of farming

**Solution:** The average age of UK farmers is 59; hence, as more farmers approach retirement age, the food industry is reliant on the younger generation to take on the challenges of the industry. The lack of interest displayed by the younger generation is explained by the stigma around farming, as it is perceived that a career in agriculture requires hard labour and tedious work for little reward. It is crucial to improve the image of agriculture and farming so that more young people think of it as a viable and potentially lucrative career option (Agricultural Recruitment Specialists, 2022). Already organisations such as Barclays are encouraging the younger generation to go into farming by launching their #FarmTheFuture campaign (Farming Online, 2018). Similarly, the government has recently invested in doctoral programmes focussed on addressing the issues in the UK food system, which allows young people to learn more about the challenges in the industry and to contribute to resolving them. Despite these efforts, the government needs to implement more long‐term solutions to ensure ongoing interest and involvement in agriculture are maintained in the years to come. Through partnerships, with schools and universities, more agri‐food subjects can be core parts of the curriculum (Agricultural Recruitment Specialists, 2022). As well as working with farmers to create apprenticeships and providing agri‐food school trips throughout the compulsory educational period of young people's lives. Exposing young people to these subjects at an early age can support the development of problem‐solving skills on top of increasing overall interest in agriculture and food systems.

These are just a few examples of the many existing solutions; however, the solutions implemented by decision‐makers will be determined by factors such as financial capabilities, feasibility and time pressures. Furthermore, the simplicity and concise way in which the different solutions suggested in this paper have been discussed do not reflect the complexity and time required to implement these changes.

## 5 CONCLUSION

In the wake of the COVID‐19 pandemic, the UK is in a unique predicament. Critical Brexit negotiations related to the food supply chain continue amidst a global pandemic that completely undermines what constitutes a resilient food system. The UK must transform and adapt its food system to meet the global standards of sustainability, as well as the postpandemic demands of consumers and stakeholders. Although the UK's food system may not face the same problems as other nations, the UK can take inspiration from states such as Sweden, Finland and Japan which are thought to be top performers in food sustainability in 2021, especially in their efforts to provide nutritional food and manage food waste. Ideally, the future UK food system would consist of a combination of short and longer food supply chains and the balanced involvement of urban and rural agriculturalists in production processes.

In regard to new regulations, it is difficult to envision what new policies and regulations will look like as these take time to develop. These considerations have to be made based on the current economic and social climate resulting from the pandemic. However, any new regulations should be clear and effective in providing labour, economic and advisory support for producers and agri‐businesses for the various challenges which they may encounter during this transitional trial period. At best, such new regulations will increase the viability of affordable domestically grown produce – both increasing food security as well as British farmers' presence in the global market.

With this in mind, a strong case can be made that the UK is not as developed as it may have been previously perceived. Its core sectors fail to meet the global standards set by the SDGs. While on the periphery the UK food system may not reflect the critical condition of the whole sector, but domestically the impacts of having an underdeveloped food system are being felt by British consumers on a local and regional scale. The UK's process of reframing and decoupling from eurocentrism may be slow and extensive as it is deeply ingrained in the culture, knowledge and values of Britain.

As identified in the review, there is a lack of research on the UK's food system. Therefore, this paper begins the line of research by focussing on a postpandemic and post‐Brexit UK food system despite the uncertainty surrounding how changing dynamics will influence the country's approaches and actions. Further contemporary research will document, critique, advise, influence decision‐making and help shape the progression of the UK's food sector. More importantly, future literature concerning the UK's food system will act as a tool to hold decision‐makers, that is Defra, and the government, accountable in the continuation of the journey towards sustainability. There should also be further research into understanding how policy can hold supermarkets accountable for their food waste as well as how to increase supermarkets' responsibility to help to resolve food insecurity in local communities. This is pertinent as food waste and food insecurity are issues that remain unresolved.

## FUNDING INFORMATION

No funding was received to support this manuscript.

## CONFLICT OF INTEREST

The authors report no conflict of interest.

## Data Availability

Data derived from public domain resources.
